# The C57BL/6J Mouse Exhibits Sporadic Congenital Portosystemic Shunts

**DOI:** 10.1371/journal.pone.0069782

**Published:** 2013-07-23

**Authors:** Cristina Cudalbu, Valérie A. McLin, Hongxia Lei, Joao M. N. Duarte, Anne-Laure Rougemont, Graziano Oldani, Sylvain Terraz, Christian Toso, Rolf Gruetter

**Affiliations:** 1 Laboratory for Functional and Metabolic Imaging (LIFMET), Center for Biomedical Imaging (CIBM), Ecole Polytechnique Fédérale de Lausanne (EPFL), Lausanne, Switzerland; 2 Département de l’Enfant et de l’Adolescent, Unité de Gastroentérologie, Hépatologie et Nutrition, Hôpitaux Universitaires de Genève (HUG), Geneva, Switzerland; 3 Faculty of Medicine, University of Geneva (UNIGE), Geneva, Switzerland; 4 Department of Radiology, University of Lausanne (UNIL), Lausanne, Switzerland; 5 Service de Pathologie Clinique, Hôpitaux Universitaires de Genève (HUG), Geneva, Switzerland; 6 Transplantation Division, Department of Surgery, University of Geneva Hospitals (HUG), Geneva, Switzerland; 7 Department of Surgery, University of Pavia, Pavia, Italy; 8 Unité de radiologie abdominale, Service de Radiologie, Hôpitaux Universitaires de Genève (HUG), Geneva, Switzerland; 9 Department of Radiology, University of Geneva (UNIGE), Geneva, Switzerland; King’s College London, United Kingdom

## Abstract

C57BL/6 mice are the most widely used strain of laboratory mice. Using *in vivo* proton Magnetic Resonance Spectroscopy (^1^H MRS), we have repeatedly observed an abnormal neurochemical profile in the brains of both wild-type and genetically modified mice derived from the C57BL/6J strain, consisting of a several fold increase in cerebral glutamine and two fold decrease in myo-inositol. This strikingly abnormal neurochemical “phenotype” resembles that observed in chronic liver disease or portosystemic shunting and appeared to be independent of transgene, origin or chow and was not associated with liver failure. As many as 25% of animals displayed the abnormal neurochemical profile, questioning the reliability of this model for neurobiology. We conducted an independent study to determine if this neurochemical profile was associated with portosystemic shunting. Our results showed that 100% of the mice with high brain glutamine displayed portosystemic shunting by concomitant portal angiography while all mice with normal brain glutamine did not. Since portosystemic shunting is known to cause alterations in gene expression in many organs including the brain, we conclude that portosystemic shunting may be the most significant problem associated with C57BL/6J inbreeding both for its effect on the central nervous system and for its systemic repercussions.

## Introduction

In the last three decades, mouse models have contributed to enormous advances in characterizing the molecular underpinnings of disease through the use of transgenesis and other approaches. The C57BL/6 strain is one of the most widely used for both transgenesis and environmental exposure experiments [1], [2], [3], [4], [5]. Low tumor incidence is one of its many advantages, unlike the high frequency in early mouse-based experiments (http://www.informatics.jax.org, http://www.harlan.com). However, several phenotypes (e.g. microphtalmia, hydrocephalus, and other gene-based anomalies) suggestive of inbreeding have been reported [6], [7].

Our facility (CIBM) is a regional imaging center for *in vivo* proton Magnetic Resonance Spectroscopy (^1^H MRS) and Imaging (MRI). As such, we routinely perform *in vivo* ^1^H MRS measurements on more than one thousand wild-type and transgenic animals originating from different laboratories and suppliers. Since establishing the CIBM (Centre d’Imagerie Biomedicale) in 2005, we have repetitively observed an abnormal neurochemical pattern consisting of increased cerebral glutamine (Gln) concentration and decreased myo-inositol (Ins) concentration in the brains of both wild-type and genetically modified mice, all of which were derived from the C57BL/6J strain. Gln and Ins were the only substantial changes in the neurochemical profile, consisting of 20 brain metabolites. This neurochemical “phenotype” was reminiscent of what has been described in patients with chronic liver disease [8], [9], [10]. The findings appeared to have a high incidence and to be independent of transgene, origin, chow, and type of experiment.

^1^H MRS spectra displaying high Gln and low Ins are typically attributed to liver failure or to portosystemic (PS) shunting [8], [9], [10], [11], [12], [13], [14], [15]. In the absence of any sign of liver failure, we hypothesized that the mice with elevated cerebral Gln measured *in vivo* by ^1^H MRS would have portosystemic shunts, a congenital anomaly well described in other mammalian species [16], [17], [18], [19]. Therefore, the aim of the present study was to determine if the C57BL/6J mice with the observed abnormal neurochemical profile showed evidence of portosystemic shunting.

## Materials and Methods

### Animals

All animal experiments were conducted according to federal and local ethical guidelines, and the protocols were approved by the local regulatory body of the Canton Vaud, Switzerland (EXPANIM (Expérience sur animaux) – SCAV, Département de la sécurité et de l’environnement, Service de la consommation et des affaires vétérinaires).

All our animals (wild-type and genetically modified) were provided by official suppliers or different academic or commercial animal facilities nationwide. For the purpose of the present study we are presenting only some of the “High Gln” mice identified in our laboratory (total of n = 23 mice). All the mice were C57BL/6J or back-crossed with this strain.

### ^1^H MRS Measurements

*In vivo* serial proton localized spectra were measured during post natal day (P) 10, 20, 30, 60, 90 to control for any developmental effect. Measurements were performed in the cortex of “Normal Gln” mice n = 11 and “High Gln” mice n = 11 (6 females and 5 males) (volume of interest (VOI) of 0.8×4×1.2 mm^3^,) at all developmental time points. In adult animals aged 4 (n = 12, 7 males) and 12 months (n = 7, 5 males), the striatum (VOI of 2×2×2 mm^3^), hippocampus (VOI of 1.2×1.6×2.2 mm^3^) and cortex (VOI of 0.8×4×1.2 mm^3^) were measured individually. Anesthesia was maintained for the length of the procedure using 1.3±0.2% of isoflurane in oxygen. Body temperature was measured with a rectal thermosensor and maintained at 36.5±0.5°C by warm water circulation. Respiration rate was monitored by a small-animal monitor system (SA Instruments Inc., New York, NY, USA). All data were acquired on a 14.1T/26 cm system (Varian/Magnex Scientific) using a home-built 14 mm×21 mm quadrature coil as RF transceiver and an ultra-short-echo time SPECIAL spectroscopy sequence (echo time (TE) = 2.8 ms, repetition time (TR) = 4 s, 400 scans) [20]. The static field was shimmed by FASTMAP [21]. After first and second order shimming, the typical linewidth of water resonance at TE = 2.8 ms was 18–23 Hz.

Metabolite concentrations were estimated using LCModel (http://s-provencher.com), combined with a simulated basis-set of metabolites and the spectrum of macromolecules measured *in vivo* [22], [23]. Absolute metabolite concentrations were obtained using unsuppressed water signal as a reference. The Cramér-Rao lower bounds (CRBs)) were calculated by LCModel as a measure of the reliability of the metabolite estimates. Using in vivo ^1^H MRS at ultra-short echo time we were able to reliable quantify 20 brain metabolites, with CRBs below 20%, involved in: myelination/cell proliferation (phosphocholine (PCho), glycerophosphocholine (GPC), phosphoethanolamine (PE), N-acetylaspartate (NAA), N-acetylaspartylglutamate (NAAG)), energy metabolism (glucose (Glc), lactate (Lac), creatine [5], phosphocreatine (PCr), alanine (Ala)), osmoregulation (taurine (Tau), myo-inositol (Ins)), neurotransmitter metabolism (glutamate (Glu), glutamine (Gln), aspartate (Asp), γ-aminobutyrate (GABA), glycine (Gly)) and antioxidants (ascorbate (Asc), glutathione (GSH)).

### Blood Measurements

Plasma ammonia and glutamine concentrations were measured from all animals using an Analox GM7 analyzer (Analox Instruments, London, UK) as previous published [24]. Assessment of liver function was performed by measuring plasma aspartate amino transferase (AST), alanine amino transferase (ALT) and total bilirubin using a Reflotron Plus analyzer (Roche Diagnostics GmbH, Mannhein, Germany). AST was considered normal when ranging between 54 to 298 (U/I), ALT when ranging between 38 to 106 (U/l), and bilirubin when <0.5 mg/dl (http://www.lar.iastate.edu, www.medical-solution.ch – Reflotron manual).

### Breeding

One high glutamine (Gln) female and male was used to obtain two litters of offspring. All offspring (n = 9) were furthermore subject to only *in vivo* ^1^H MRS measurements as previously described.

### Portal Angiography

Five “High Gln” (n = 3 at 4 months and n = 2 at 12 months, males) and five “Normal Gln” (n = 3 at 4 months, males and n = 2 at 12 months, females) mice underwent ^1^H MRS measurements prior to portal angiography, to confirm the abnormal neurochemical pattern. Mice were anesthetized using isoflurane (3% for induction and 2% thereafter, 1L/min O_2_ flow). Following a midline incision, the mesentery was overturned to the left side of the abdomen in order to best expose the inferior mesenteric vein. The vein was isolated and encircled with two 7–0 silk ties, then a heparin-flushed 26-French catheter was introduced into the vein and secured with the silks. The tip of the catheter was placed between the gastro-splenic and the duodeno-pancreatic veins.

Portal angiographies were performed on a digital subtraction angiography system equipped with a 40 × 48 cm flat panel detector (Allura Xper FD 20; Philips Medical System, Best The Netherlands). The mice were placed in the supine position on the angiography table. The 26-F catheter in the portal vein was attached through a 26-F connecting tube to an infusion pump (Module DPS Orchestra; Frenesius Vial, Brezins, France). Standard postero-anterior angiograms were obtained during continuous injection of iodinated contrast media (350 mg iodine/ml of iohexol, Accupaque; GE Healthcare, Oepfikon, Switzerland), Cork, Ireland) with the following image parameters: tube voltage, 120 kV; modulated tube current; inherent filtration, 2.7 mm aluminium; pulse width, 6 ms; system dose, 0.36 µGy per pulse; focal spot, 0.7 mm; tube detector distance, 0.4 m; matrix size, 1024×1024; zoom factor, 5x; field of view, 7×4 cm; acquisition time, 15 s; delay time, 2 s. Then, a 3D rotational computed tomography (CT) was acquired with the following parameters: a total acquisition angle of 222°, projection increment of 0.8°. The CT images were reconstructed and analyzed on a dedicated workstation (Xper CT; Philips Medical System, Best The Netherlands). In order to optimize contrast media injection, we performed successive portal angiographies with different flow rate (75 mL/h; 150 mL/h; 300 mL/h; 600 mL/h) on two test animals. The optimal flow rate of 150 mL/h (corresponding to 0.042 mL/s) was used in the “Normal Gln” group (n = 5) and in the “High Gln” group (n = 5).

### Histology

Livers were harvested following portal angiography (n = 5 for “High Gln” mice and n = 5 for “Normal Gln” mice). To control for injection artifacts (i.e. modifications of the liver parenchyma restricted to the lobules, which did not impact assessment of portal tract and centrilobular vein), we also harvested livers from two 4 months old “High Gln” female mice and two 4 months old “Normal Gln” female mice (previously confirmed by ^1^H MRS) having not undergone portal angiography.

All tissues were fixed in 10% buffered formalin overnight at room temperature, then paraffin-embedded. For routine histological examination, 3 µm-thick sections were stained with haematoxylin-eosin (H&E), Masson’s trichrome, and a reticulin stain. Immunohistochemistry was performed on paraffin-embedded sections mounted on positively charged slides, using an automated DAKO immunostainer (DakoCytomation, Glostrup, Denmark). The following primary antibodies were applied: CD31 (PECAM-1, M-20, sc-1506, goat polyclonal, Santa Cruz Biotechnology, Inc., Heidelberg, Germany), and D2–40 (Podoplanin, goat polyclonal, R&D Systems, Abingdon, UK). First, for antigen retrieval, deparaffinized and rehydrated sections were treated using an electric pressure cooker for CD31 (Pascal Tris EDTA buffer, pH7.0, for 30 seconds) and a microwave for D2–40 (citrate buffer, pH6.0, for 10 minutes). Then the sections were mounted in the DAKO autostainer, covered with blocking peroxidase for 5 minutes. Slides were incubated for 60 minutes with the diluted antibody (CD31 1:200; D2–40 1:1000). This step was followed by applying the labelled rabbit anti-goat HRP method (DAKO; 1∶20, 30 minutes). DAB (DAKO) was used as a chromogen.

Apart from routine liver architecture, portal tract, centrilobular vein, and hepatocyte evaluation, the following were also assessed: relative sizes of portal veins and lymphatics, total numbers of portal tracts and lymphatic vessels, and mean lymphatic profiles per portal tracts.

### Statistical Analyses

All the results are presented as mean ± SD of n mice, unless otherwise indicated. A two-way ANOVA followed by the Bonferroni’s multi-comparison post-test was used to compare the concentration of neurochemicals. ANOVA during development was performed for each neurochemical in 2 groups×5 time points (ages from P10 to P90), whereas in adult mice was performed for each neurochemical at each age in 2 groups×3 brain areas. Statistical analyses were performed using Prism 5.03 (GraphPad, La Jolla CA USA).

## Results

**Figure 1:**
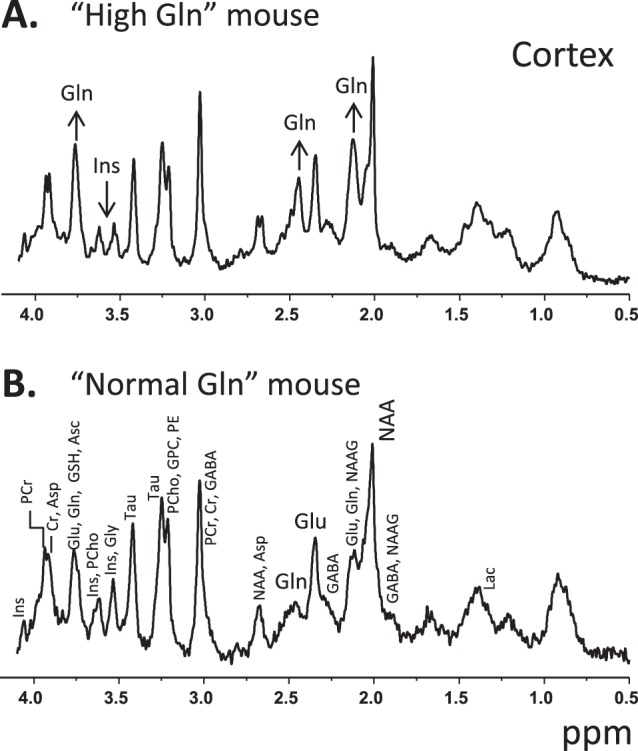
Representative *in vivo* spectra acquired at 14.1T in the cortex (VOI = 0.8×4×1.2 mm^3^) of “High Gln” (A) and “Normal Gln” (B) C57BL/6J mice. The increase of Gln and decrease of Ins in the cortex of the “High Gln” mouse is visually apparent. Only those metabolites displaying a concentration change are labeled (i.e. Gln and Ins) for the “High Gln” spectrum.

**Figure 2:**
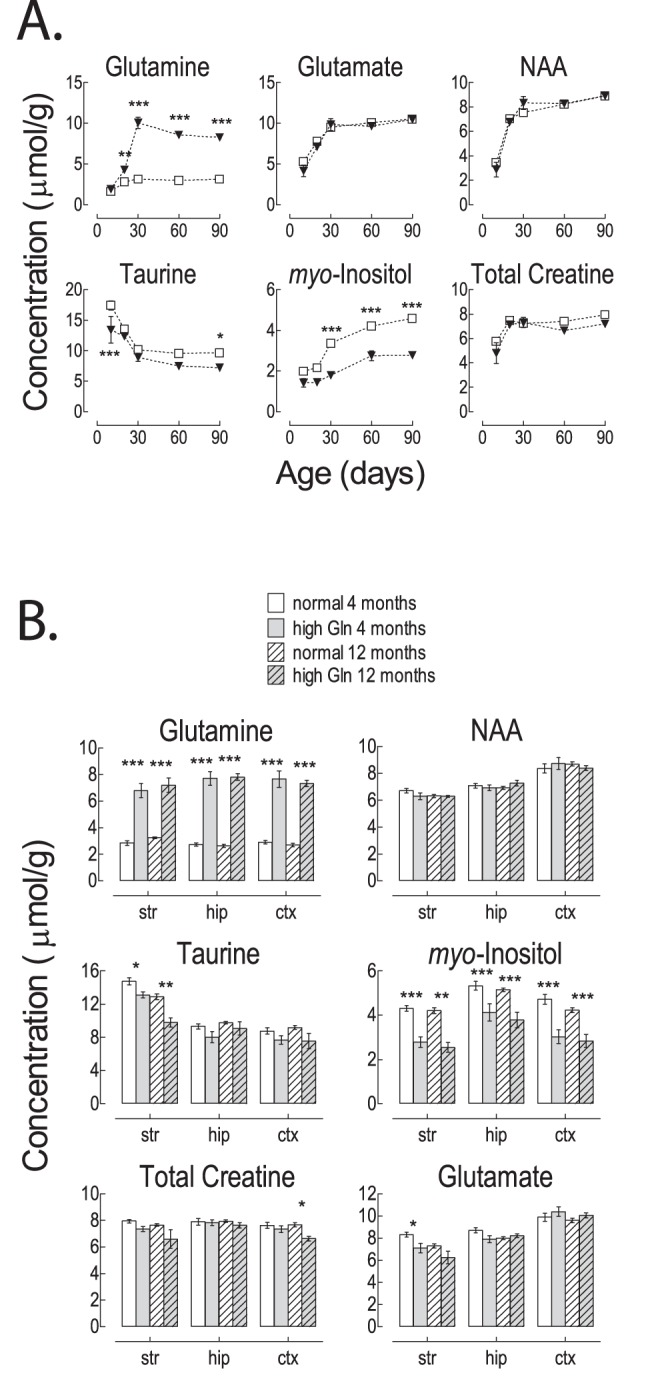
*In vivo* ^1^H MRS results obtained in the brain of “High Gln” and “Normal Gln” C57BL/6J mice. A) Evolution of brain metabolite concentrations during mouse development at P 10, 20, 30, 60 and 90; open squares indicate “Normal Gln” mice and triangles indicate “High Gln” mice; and B) metabolite concentrations in the striatum (str), hippocampus (hip) and cortex (ctx) of “High Gln” and “Normal Gln” C57BL/6 mice at 4 and 12 months of age. Two-way ANOVA was performed at 5 developmental time-points (ages P10 to P90) comparing the “High Gln” mice to “Normal Gln” mice for each metabolite. Statistically significant differences for Gln, Tau and Ins between “High Gln” and “Normal Gln” mice (df = 1, F value between 23.6 and 625) are marked *(p<0.05), **(p<0.01) and ***(p<0.001). The age comparison showed statistical differences for all plotted metabolites (p<0.0001, df = 4, F value between 19.2 and 139) (not shown). We observed statistically significant age-dependent differences between groups for Ins and Gln (p = 0.002 and p<0.0001 respectively, df = 4, F value 4.47 and 66.7, respectively) (not shown). In adult mice two-way ANOVA was performed for each neurochemical at each age (4 and 12 months) in 2 groups (“High Gln” vs “Normal Gln” mice)×3 brain areas. Statistically significant differences for Gln, Glu, Tau, Ins and tCr between “High Gln” and “Normal Gln” mice (df = 1, F value between 14 and 519) are marked *(p<0.05), **(p<0.01) and ***(p<0.001). Additionally, the brain regions comparison showed statistical differences for some of the plotted metabolites (Glu, NAA, Tau, Ins p<0.001, df = 2, F value between 10 and 87) (not shown). Lac: lactate, GABA: γ-aminobutyrate, NAAG: N-acetylaspartylglutamate, NAA: N-acetylaspartate, Gln: glutamine, Glu: glutamate, Asp: aspartate, Cr: creatine, PCr: phosphocreatine, PE: phosphoethanolamine, PCho: phosphocholine, GPC: glycerophosphocholine, Tau: taurine, Ins: myo-inositol, Gly: glycine, GSH: glutathione, Asc: ascorbate.

The ultra-short echo time sequence combined with the availability of ultra-high magnetic field (14.1T) yielded ^1^H spectra with excellent signal-to-noise ratio (SNR) and with a clear separation of the Gln and glutamate (Glu) peaks (Figure 1). In adult mice the Gln concentration was considered to be normal when ranging between 2.7–3.8 µmol/g, as previously described [25], [26] and illustrated in Figures 1 and 2. Additionally, adult “High Gln” mice displayed Gln concentrations ranging from 5.5 to 12 µmol/g.

To determine whether the neurochemical changes in Gln and Ins could be ascribed to developmental fluctuations, we performed serial *in vivo* ^1^H MRS measurements in the cortex of developing mice from post-natal day (P) 10 to P90 (Figure 2A). In brief, our findings show that cerebral Gln concentrations rise very early in the “High Gln” pup, reaching a plateau in the adult. A 20% increase in Gln concentration was noticeable as early as P10, reaching a 50% increase at P20 (p<0.01) in the “High Gln” mice compared to age-matched animals coming from the same supplier. At P30 the “High Gln” mice showed a ∼3 fold elevation of cerebral Gln compared to control mice (10.0±1.7 µmol/g for “High Gln” mice and 3.1±0.3 µmol/g for “Normal Gln” mice, p<0.001) with no further increase at P90. Likewise, a 28% decrease in Ins was observed in the pup at P10, continuing further until P90 (i.e. 50% decrease at P30, p<0.001) in “High Gln” mice. Ins concentration remained constant thereafter, in keeping with Gln findings. Taurine, another important brain osmolyte, is the only other metabolite which displayed variation, also showing variation from the norm in the very small pups (25% decrease) in “High Gln” mice, which reached significance at P10 and P90 (p<0.05). No statistically significant modifications in the concentration of other brain metabolites were observed (Figure 2A).

To determine whether the increase of Gln was region specific, we performed *in vivo* neurochemical profile assessments of the striatum, hippocampus and cortex in 4- and 12-month old mice (Figure 2B). High Gln (significant 3 fold increase compared to controls, p<0.001) and low Ins concentrations (significant 30% decrease) were apparent in all brain regions investigated at 4 and 12 months of age in the “High Gln” mice. The abnormal neurochemical pattern observed in cortex, striatum and hippocampus of “High Gln” adult mice was similar to the results obtained during brain development. The other metabolites did not show any significant temporal or spatial fluctuations, except for a small decrease of other brain osmolytes, i.e. Tau (20%) in the striatum and tCr (10%) in the cortex of “High Gln“ mice at 12 months of age.

To determine the presence of a systemic phenotype, we performed serial weight and biochemical measurements. There was no significant difference in mean weight between the “High Gln” and “Normal Gln” mice at any time point: “High Gln” at 4 months weighed 30±3 g and “Normal Gln” weighed 29±3 g. No statistical differences were observed for plasma ammonia (270±40 µM for “High Gln” mice and 270±60 µM for “Normal Gln” mice, p = 0.9) and plasma glutamine concentrations (0.90±0.06 mM for “High Gln” mice and 0.88±0.12 mM for “Normal Gln” mice, p = 0.8). As the aforementioned changes are a hallmark of hepatic encephalopathy in humans [8], [9], [10], [11], [12], [13], we assessed liver function in “High Gln” and “Normal Gln” mice. Total bilirubin, aspartate amino transferase (AST) and alanine amino transferase (ALT) were within normal limits for all animals (total bilirubin was below 0.5 mg/dl for all investigated animals). AST and ALT remained within the normal range but displayed a higher variability in the “High Gln” mice (AST: 272±169 U/l and ALT: 82±56 U/l) compared to “Normal Gln” mice (AST: 102±52 U/l and ALT: 22±6 U/l) (p = 0.1 for both AST and ALT).

To characterize the heritability pattern of this trait, a “High Gln” male and “High Gln” female were crossed, yielding two litters (n = 9). None of the pups showed the characteristic abnormal neurochemical profile (data not shown) by ^1^H MRS, and therefore no further measurements were performed on these animals.

**Figure 3:**
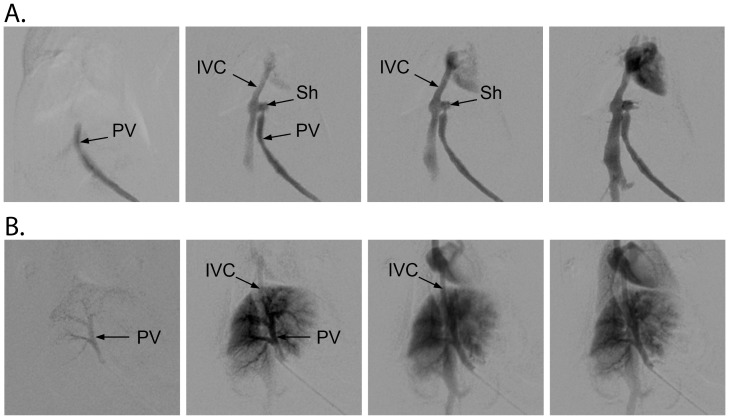
Representative angiographies obtained in a “High Gln” mouse (A) and in a “Normal Gln” (B) C57BL/6J mouse. Normal filling of the portal tree is visible in B. In A, injection in the superior mesenteric vein leads to immediate filling of the inferior vena cava. Inferior vena cava (IVC), portal vein (PV), shunt (Sh).

To assess the presence of portosystemic shunts, portal angiograms were obtained in all animals (Figure 3), after performing ^1^H MRS measurements to confirm the abnormal neurochemical pattern. The portal vein was patent in all animals, with hepatopetal flow and a mean diameter of 1.3±0.2 mm (1.1–1.6 mm). There were no angiographic signs of portal hypertension. In “Normal Gln” mice, contrast media flowed from the portal vein immediately into the portal branches of the liver (Figure 3B). After the parenchymal phase, the contrast entered the suprahepatic inferior vena cava with a small retrograde filling of the intrahepatic inferior vena cava. Conversely, in “High Gln” mice, the contrast medium flowed from the portal vein directly into the suprahepatic inferior vena cava, without enhancement of the intrahepatic portal veins or the liver parenchyma (Figure 3A). The CT images indicated a shunt between the portal vein and the inferior vena cava in all cases (n = 5). The shunt was visible as a short segment that ran perpendicular to both the portal vein and inferior vena cava within the left side of the liver, consistent with a patent ductus venosus. Mean shunt length was 1.6±0.4 mm (1.2–2.2 mm). In two animals of this group, we also detected an early enhancement of the left atrium, suggestive of an atrial septal defect.

**Figure 4:**
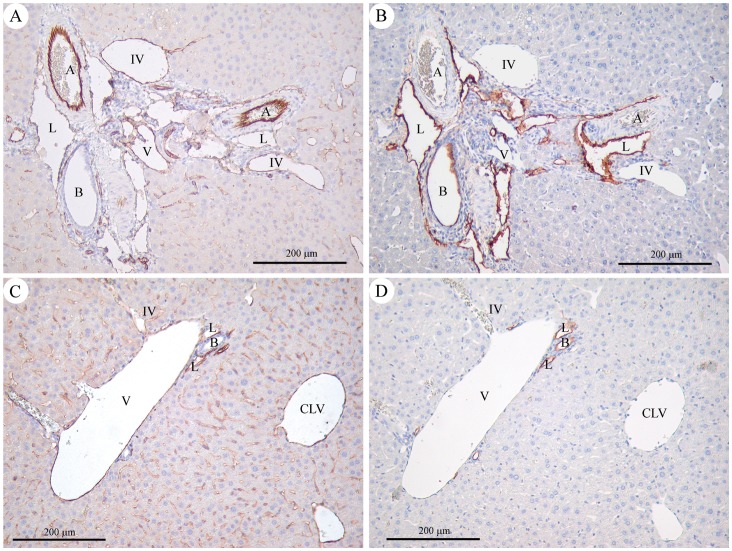
Representative immunohistochemistry findings in a “High Gln” mouse (A, B) and in a “Normal Gln” mouse (C, D). Original magnification 100x. A and C: CD31 immunostaining highlights endothelial cells. In the “High Gln” mouse (A), the hepatic artery branch is of normal size, similar to that of the interlobular bile duct; the portal vein is small and hypoplastic, and the inlet venules are dilated. In the “Normal Gln” mouse (C), the portal vein is large, with a normal size ratio to the interlobular bile duct (the hepatic artery branch is not seen in this section). The inlet venule is thin. B and D: D2–40 expression confirms the lymphatic nature of the dilated channels at the periphery of the portal tracts in the “High Gln” mouse (B), being selectively reactive in lymphatic endothelial cells, contrary to arterial and venous endothelial cells. Of note, D2–40 (podoplanin) reactivity is also seen in bile duct epithelium. Abbreviations: A = hepatic artery; B = interlobular bile duct; CLV = centrilobular vein; IV = inlet venule; L = lymphatic vessel; V = portal vein.

PS shunts are associated with subtly abnormal liver histology [19], [27]. Portal vein branch hypoplasia is the most characteristic finding [19], [27]. We therefore assayed the histology of “Normal Gln” mice (n = 5 after angiography and n = 2 without angiography) and “High Gln” mice (n = 5 after angiography and n = 2 without angiography). No architectural remodeling, or portal tract fibrosis were observed. Lobular organization and hepatocyte structure were normal in appearance. Portal vein anomalies were observed exclusively in the “High Gln” mice. They consisted of small, hypoplastic or absent portal veins in portal tracts, in association with frequent inlet veinule dilatation. Portal vein anomalies were seen in all “High Gln” mice, in 36% to 80% of portal tracts (80% [16/20]; 44.4% [16/36]; 36% [18/50]; 42.5% [34/80]; 44.8% [26/58]). Medium-sized portal tracts were mostly affected, whereas control mice showed large intrahepatic portal veins in all assessed portal tracts. Further, lymphatic channel ectasia was observed and confirmed after D2–40 immunohistochemistry (Figure 4). It was more prominent in the “High Gln” mice, but was also observed in the controls. Mean lymphatic profile count per portal tracts did not differ significantly (“High Gln” mice: 1.81 to 4.3 lymphatic profiles/portal tract; controls: 1.5 to 2.44) (p = 0.097). Centrilobular veins were unaffected in both “High Gln” and “Normal Gln” animals.

## Discussion

This study shows that a high fraction of C57BL/6J mice present an abnormal neurochemical profile consisting of elevated cerebral Gln concentrations and decreased cerebral Ins (“High Gln” mice), and that these changes are associated with the presence of congenital portosystemic shunts.

To the best of our knowledge, this is the first report describing an abnormal neurochemical profile identified using *in vivo* ^1^H MRS in a large proportion of wild-type and genetically modified C57BL/6J mice. However, the incidence of this profile was variable. Findings were independent of transgene, type of investigation, chow or origin.

Note the excellent agreement with previous studies of regional and developmental changes (Figure 2) in the murine neurochemical profile [25], [26]. For example, NAA concentration in the cortex of adult “Normal Gln” mice was 8.6±0.7 µmol/g in perfect agreement with the value of ∼8.5 µmol/g previously reported [25], [26].

The abnormal neurochemical profile consisted of increased cerebral Gln concentration by an average of 3 fold and 50% decreased cerebral Ins affecting all brain regions. It has to be emphasized that a 3 fold increase in Gln is well in excess of any change considered statistically significant in a given mouse for all of the studies performed at our center.

Therefore, this unique phenotype is far from a variation of the norm. This characteristic profile is typical of hepatic encephalopathy associated with chronic liver disease and portosystemic shunting [8], [9], [10], [11], [12], [13], [15]. Here, we show that 100% of “High Gln” animals subjected to portal angiography displayed intrahepatic portosystemic shunts, in contrast to the “Normal Gln” mice. Liver histology revealed hypoplastic portal branch segments consistent with descriptions in dogs and humans [19], [27].

The commonly accepted pathophysiology of central nervous changes in liver disease and portosystemic shunting is that portal blood bypasses the liver, thereby reaching the systemic circulation without undergoing the necessary transformation in the liver. Thus, exogenous and endogenous substances such as ammonia enter the systemic circulation directly. In the central nervous system, ammonia is detoxified into Gln via the astrocyte-specific enzyme glutamine synthethase [15], [24], [28], [29]. The end-result of CNS ammonia detoxification is elevated Gln concentration in astrocytes and efflux of other osmolytes [8], [9], [10], [11], [12], [13], [14], [15]. We therefore conclude that the abnormal neurochemical profile detected in the C57BL/6J mice is most likely due to portosystemic shunting-induced CNS ammonia detoxification. The associated decrease in Ins and other osmolytes is consistent with osmoregulation [8], [9], [10], [11], [12], [13], [14], [15]. In the present study, there was a noticeable decrease of other brain osmolytes (Tau and tCr) in older animals, suggestive of ongoing osmoregulation and consistent with published findings in models of chronic liver disease and in patients [8], [9], [10], [15].

High cerebral Gln concentration is detectable *in vivo* only by ^1^H MRS. No, biochemical or physical measurements performed allowed to distinguish “High Gln” from the “Normal Gln” mice. This raises the important question of how to screen for this defect. At present, we are not aware of an affordable and practical alternative to *in vivo* ^1^H MRS scanning. Plasma ammonia does not seem to be sensitive enough since this measurement was within the normal range for most of our animals, also observed in dogs and humans [19], [30], [31].

Congenital PS shunts are characterized by similar pathophysiology and histology, whether intra- or extrahepatic. The shunts identified in our animals resemble incomplete ductus venosus (DV) closure, typical for intrahepatic shunts [32]. Congenital PS shunts have been described in other mammals (cats, dogs) and humans [16], [17], [18], [19], [27], [32]. However, the cause of this developmental defect remains unclear: in Irish wolfhounds, patency of the DV appears to be a digenic trait [19], while in Cairn terriers non-closure of the DV appears to follow a more complex mode of inheritance [19]. The serendipitous finding that in the Ahr−/− mouse model (aryl hydrocarbon receptor deficient mice), all null animals show patency of the DV [33] has put forward the Ahr-ARNT-CYP1 signaling cascade as a possible candidate in embryonic vascular remodeling. However, there are two reasons why this gene is unlikely to be the sole culprit in the C57BL/6J mouse: a) the inheritance pattern differs and b) the Ahr−/− mouse displays a number of other vascular anomalies [33] not present in the C57BL/6 mouse. In our study offspring of “High Gln” parents did not show any neurochemical anomalies, suggesting a non-mendelian inheritance pattern. Furthermore, environmental influences appear to be unlikely as the defect was identified in a large number of wild-type and transgenic animals hosted in more than 5 animal facilities. Epigenetic modifications are currently accepted to be at the root of seemingly erratic inheritance patterns [34], [35], [36], something we aim to address in further studies. Finally, no other strain of mice investigated in our center to date displayed such a neurochemical profile. In the last 7 years we have performed ^1^H MRS in more than one thousand mice originating from at least 7 different mice strains (including NMRI, BALB/c, ICR CD1, NUDE, NOD-SCID, SV 129, C57BL/6). Therefore, we propose that akin to what is seen in purebred dogs, inheritance of intrahepatic PS shunt is complex, and possibly limited to the C57BL/6 strain, the most commonly used strain of laboratory mice.

It is interesting to note that the same abnormal neurochemical profile identified using *in vivo* ^1^H MRS (increased brain Gln and decreased brain Ins) was reported in a significant proportion of wild-type and genetically modified C57BL/6 mice in a study of a mouse model of Huntington’s disease [37], although the underlying cause was not identified.

We observed that as many as 25% (or even higher) of C57BL/6J animals enrolled in a given study conducted in our facility displayed an abnormal neurochemical profile in all regions of the brain, and that this finding existed in animals with congenital PS shunts. The frequency of this characteristic neurochemical profile, typically attributed to PS shunting or chronic liver disease, raises the question of the reliability of this model for neurobiology in the absence of appropriate tools to screen the animals.

The implications of the current findings are likely far-reaching: in humans, PS shunting is associated with many systemic changes, namely cardiac, renal, hepatic and pulmonary [32]. First, it has recently been shown in PS shunting that the expression of a variety of genes is altered in the central nervous system including that of neurotransmitter receptors, transporters and members of secondary messenger signal transduction [38]. PS shunting has also been shown to display significant abnormalities in the biology of inflammation [39]. Second, there is ample evidence of altered gene expression in other organs in PS shunting, such as: skeletal muscle, pancreas, peripheral tissues and liver [38], [40], [41], [42], [43], [44], [45], [46].

We conclude that, although congenital anomalies associated with the inbreeding of C57BL/6 have already been identified (ocular defects, genetic polymorphisms among substrains, behavioral differences among substrains [1], [2], [3], [4], [5], [6], [7]), PS shunting may well be the most significant. Using ^1^H MRS of mouse brain or an appropriate, as of yet unknown, other screening method may allow to screen for PS shunted animals, and thus to control for the systemic effects of PS shunting in studies based on the C57BL/6 strain and possibly derived strains.
